# Equity impact of HPV vaccination on lifetime projections of cervical cancer burden among cohorts in 84 countries by global, regional, and income levels, 2010–22: a modelling study

**DOI:** 10.1016/j.eclinm.2024.102524

**Published:** 2024-03-11

**Authors:** Kaja Abbas, Katelyn Jison Yoo, Kiesha Prem, Mark Jit

**Affiliations:** a London School of Hygiene & Tropical Medicine, London, UK; b School of Tropical Medicine and Global Health, Nagasaki University, Nagasaki, Japan; c Johns Hopkins Bloomberg School of Public Health, Baltimore, USA; d Health, Nutrition and Population, World Bank, South Korea; e Saw Swee Hock School of Public Health, National University of Singapore, Singapore; f School of Public Health, The University of Hong Kong, Hong Kong SAR, People's Republic of China

**Keywords:** Cervical cancer, Human papillomavirus, Vaccine equity, Vaccine impact modelling

## Abstract

**Background:**

While human papillomavirus (HPV) vaccines have been available since 2006, the coverage has varied among countries. Our aim is to analyse the equity impact of HPV vaccination on the lifetime projections of cervical cancer burden among vaccinated cohorts of 2010–22 in 84 countries.

**Methods:**

We used WHO and UNICEF estimates of national immunisation coverage for HPV vaccination in 84 countries during 2010–22. We used PRIME (Papillomavirus Rapid Interface for Modelling and Economics) to estimate the lifetime health impact of HPV vaccination on cervical cancer burden in terms of deaths, cases, and disability-adjusted life years (DALYs) averted by vaccination in their respective countries. We generated concentration indices and curves to assess the equity impact of HPV vaccination across 84 countries.

**Findings:**

The health impact of HPV vaccination varied across the 84 countries and ranged from Switzerland to Tanzania at 2 to 34 deaths, 4 to 47 cases, and 40 to 735 DALYs averted per 1000 vaccinated adolescent girls over the lifetime of the vaccinated cohorts of 2010–22. The concentration index for the distribution of average coverage during 2010–22 among the 84 countries ranked by vaccine impact was 0.33 (95% CI: 0.27–0.40) and highlights the wide inequities in HPV vaccination coverage.

**Interpretation:**

Our findings suggested that countries with a relatively higher cervical cancer burden and thereby a relatively higher need for HPV vaccination had relatively lower coverage during 2010–22. Further, there were significant inequities in HPV vaccination coverage within the Americas, Europe, and Western Pacific regions, and in high- and low-income countries with a pro-advantaged and regressive distribution favouring countries with lower vaccine impact.

**Funding:**

Gavi, the Vaccine Alliance; 10.13039/100000865Bill & Melinda Gates Foundation.

**Research in context**

**Evidence before this study**

We searched PubMed on August 17, 2023, with the following search terms: (“HPV vaccin∗” and “equity”) to identify 76 articles, of which 14 articles were of relevance. The studies included a conceptual framework to assess HPV vaccination impact on gender equity and model-based inference of HPV vaccination on health inequity. Other studies assessed reduction of cervical cancer disparities in New Zealand; mode of HPV vaccine delivery in schools and primary care in New Zealand and Sweden; extended cost-effectiveness analysis of publicly financed HPV vaccination in China; sociodemographic correlates of HPV vaccine uptake in Norway; racial, ethnic, gender, and sexual orientation identity differences on HPV vaccination timeliness in the US; impact of including adolescent boys in the national HPV vaccination programmes in Singapore and the UK; geographical heterogeneity in HPV vaccination coverage among rural areas of Catalonia; and school factors on adolescent HPV vaccination initiation and completion in Australia.

**Added value of this study**

The current state of HPV vaccine introduction, scale-up, and coverage after more than 17 years since the first HPV vaccines being available since 2006 highlights the persistent challenges to equitable access to HPV vaccines and more broadly on fair access to vaccines in general, especially in low- and middle-income countries.

**Implications of all the available evidence**

Since the availability of the first licensed HPV vaccines in 2006, the introduction and uptake of HPV vaccination among countries have been highly inequitable to date (2022). Further, many countries with relatively higher cervical cancer burden are yet to introduce HPV vaccination in their national immunisation programmes. However, the WHO recommendation in 2022 on the one-dose schedule and projected increases in HPV vaccine supply provide a timely and favourable pathway to improve HPV vaccine equity by reducing the barriers on the programmatic, logistical, and financial fronts in delivering HPV vaccination to adolescent girls and accelerating progress towards cervical cancer elimination globally.

## Introduction

Cervical cancer is the fourth leading cause of cancer mortality and fourth most commonly diagnosed cancer among women globally, with a burden of 342,000 deaths and 604,000 new cases in 2020.^1^ Furthermore, cervical cancer is the leading cause of cancer mortality in 36 countries and the most commonly diagnosed cancer in 23 countries, with most affected countries located in sub-Saharan Africa, Melanesia, South America, and South-East Asia. The global inequities in cervical cancer burden detrimentally impact low- and middle-income countries (LMICs) the most with nearly 90% of cervical cancer mortality occurring in LMICs in 2018.^2^

As of May 2023, there are three bivalent vaccines, two quadrivalent vaccines, and one nonavalent vaccine for a total of six licensed human papillomavirus (HPV) vaccines.^3^ Bivalent and quadrivalent HPV vaccines protect against persistent infection with high-risk HPV 16/18 genotypes which cause 70% of all cervical cancers.^4^ The nonavalent vaccine provides additional protection against persistent infection with high-risk HPV types 31/33/45/52/58 which cause an additional 18.5% of HPV-positive cervical cancers.^5^

In 2008, the Strategic Advisory Group of Experts (SAGE) on vaccines and immunisation of the World Health Organization (WHO) recommended the use of HPV vaccines. The dosing regimen for HPV vaccines had changed since its initial introduction. In 2009, the WHO position paper on HPV vaccines recommended a three-dose regimen for young adolescent girls of 9 or 10 years through to 13 years.^6^^,^^7^ Clinical trials comparing the two-dose to three-dose regimen demonstrated non-inferior immune response in adolescent girls less than 15-years old.^8^, ^9^, ^10^ In 2014 based on this evidence, WHO SAGE revised its recommendation to a two-dose regimen for adolescent girls less than 15-years old while a three-dose regimen was still recommended for adolescent girls 15 years and older as well as for immunocompromised or HIV-positive individuals.^11^ In 2017, new recommendations were introduced for vaccination strategies that target girls only, both girls and boys, and multiple birth cohorts.^12^ In 2022, WHO SAGE evaluated the emerging evidence on the efficacy of one-dose regimen in comparison to two or three-dose regimens.^13^ The SAGE review concluded that a one-dose regimen likely provides comparable protection to that of a two-dose regimen. Specifically, it recommends one- or two-dose regimen for the primary target of adolescent girls aged 9–14 years, one- or two-dose regimen for young women aged 15–20 years, and two-dose regimen for women older than 21 years.

In 2018, the WHO Director General issued a global call for action to eliminate cervical cancer as a public health problem through improved coverage for HPV vaccination, high-precision screening tests, and treatment and care.^14^ The global strategy to accelerate the elimination of cervical cancer as a public health problem includes 90–70–90 targets for 2030—(i) 90% coverage of HPV vaccination among girls by 15 years of age; (ii) 70% coverage of screening among women at 35 and 45 years of age and 90% treatment of precancer lesions; and (iii) 90% coverage of treatment and care among women diagnosed with cervical cancer.

Since the first HPV vaccines became available in 2006, the introduction timeline, implementation scale-up, and uptake of HPV vaccines had varied among countries due in part attributable to the political economy, sociobehavioural determinants, and cultural beliefs. Up to 2022, less than half of the countries have included HPV vaccines in their national immunisation programmes,^15^ covering only 15% of girls in the target age for HPV vaccination in 2019.^16^ The prevalent inequities in cervical cancer burden and HPV vaccination coverage among different countries serve as useful tracers to monitor and assess the progress towards cervical cancer elimination. To compare the differential coverage among countries and corresponding impact on reduction of cervical cancer burden, we analysed the equity impact of HPV vaccination on the lifetime projections of cervical cancer burden among vaccinated cohorts of 2010–22 in 84 countries.

## Methods

### HPV vaccine impact model

The Papillomavirus Rapid Interface for Modelling and Economics (PRIME) is an HPV vaccine impact model to assess the direct health impact and cost-effectiveness of HPV vaccination of girls for prevention of cervical cancer.^17^, ^18^, ^19^ It has been endorsed by the WHO Immunization and Vaccines Implementation Research Advisory Committee (IVIR-AC) to provide a conservative estimate of the health impact and cost-effectiveness of vaccinating girls before sexual debut.^20^ PRIME has been used to inform the impact of vaccine investments by Gavi, the Vaccine Alliance, in 112 countries.^21^^,^^22^

PRIME is a static proportional impact model that estimates HPV vaccination impact of both single-age and multiple-age cohorts.^17^^,^^18^ We assessed vaccination impact in terms of reduction in cervical cancer burden (deaths, cases, and disability-adjusted life years (DALYs) averted) by estimating the reduction in age-specific cervical cancer incidence, prevalence, and mortality in direct proportion to HPV vaccine efficacy, coverage, and distribution of high-risk HPV types 16/18 (see Appendix A1 for model details). Based on the efficacy observed in vaccine trials,^23^ vaccinating adolescent girls before sexual debut fully protects them from developing cervical cancer caused by high-risk HPV types. We excluded indirect (herd) effects and cross-protection, and therefore the HPV vaccine impact estimates are conservative. This also excludes the impact on cervical cancer by vaccinating males (through indirect protection) in countries with gender-neutral programmes (mostly high-income countries).

### HPV vaccine coverage data

We used WHO and UNICEF (United Nations Children's Fund) estimates of national immunisation coverage (WUENIC) for the last dose of HPV vaccination by age 15 years among females during 2010–22, which were available for 90 countries.^15^ We excluded six countries (Andorra, Cook Islands, Palau, San Marino, Seychelles, and Saint Vincent and the Grenadines) in our analysis due to lack of data on other key inputs (such as demography) to the HPV vaccine impact model (PRIME). Among the remaining 84 countries, we used the coverage estimates for 41 high-income countries, 26 upper-middle-income countries, 13 lower-middle income, and 4 low-income countries for a total of 84 countries.

### Comparative vaccination scenarios

We estimated the lifetime impact of HPV vaccination at 14 years of age among the vaccinated cohorts of 2010–22 in 84 countries based on their reported vaccine coverage. We estimated the reduction in the lifetime burden (deaths, cases, and DALYs averted) of cervical cancer in comparison to the counterfactual scenario of no vaccination. Since the nonavalent vaccine obtained first national licensure in 2014, some (mostly high-income) countries have introduced it. However, we focused on the lifetime impact of HPV vaccination (bivalent, quadrivalent, and nonavalent vaccines) on the reduction in cervical cancer burden caused by only the high-risk HPV 16/18 genotypes (which cause 70% of all cervical cancers).

### Vaccine impact metrics

We estimated vaccine impact by country in terms of cervical cancer burden (deaths, cases, and DALYs) averted per 1000 vaccinated girls. This vaccine impact metric is not affected by vaccination coverage for any given year, considering the limitation that we estimated only the direct effects through PRIME and indirect (herd) effects are excluded.

### Equity impact analysis

Based on a critical appraisal of different methods used to measure inequalities in health, concentration index is a suggested metric for robust assessment of the socioeconomic inequalities in health.^24^ The concentration curve ranks people by socioeconomic status from the most disadvantaged to least disadvantaged, with x-axis representing the socioeconomic status and y-axis representing the cumulative percentage of health. The concentration curve will coincide with the diagonal if health is equally distributed among the socioeconomic groups. If lower socioeconomic groups have a higher concentration of poor health, then the concentration curve is below the diagonal, and vice versa—if lower socioeconomic groups have a higher concentration of good health, then the concentration curve is above the diagonal. The further the concentration curve lies from the diagonal, the greater the degree of inequality in health. The concentration index is measured by twice the area between the concentration curve and the diagonal. It is bounded between −1 and + 1, with 0 representing perfect equality and ±1 representing perfect inequality.^25^ Positive values indicate a pro-advantaged (regressive, i.e. favouring least disadvantaged socioeconomic groups—area below the line of equality) distribution of health, while negative values indicate a pro-disadvantaged (progressive, i.e. favouring most disadvantaged socioeconomic groups—area above the line of equality) distribution of health.

In our study to assess the equity impact of HPV vaccination, we generated concentration curves and indices to analyse the distribution of average coverage among the 84 countries during 2010–22. For the concentration curve—x-axis represents the country ranking from high to low vaccine impact which is the same as country ranking from high to low burden of cervical cancer (most disadvantaged to least disadvantaged), while y-axis represents the cumulative distribution of HPV vaccination coverage among the 84 countries. For the concentration index, the positive values indicate a pro-advantaged (regressive, i.e. favouring low impact countries) distribution of HPV vaccination coverage, while negative values indicate a pro-disadvantaged (progressive, i.e. favouring high impact countries) distribution of HPV vaccination.

All data were from secondary sources in the public domain, and therefore ethics approval was not required.

### Role of the funding source

The funders of this study had no role in study design, data collection, data analysis, data interpretation, or writing of the manuscript. All authors had full access to data in the study, and final responsibility for the decision to submit for publication.

## Results

### HPV vaccination coverage and impact

**Fig. 1:**
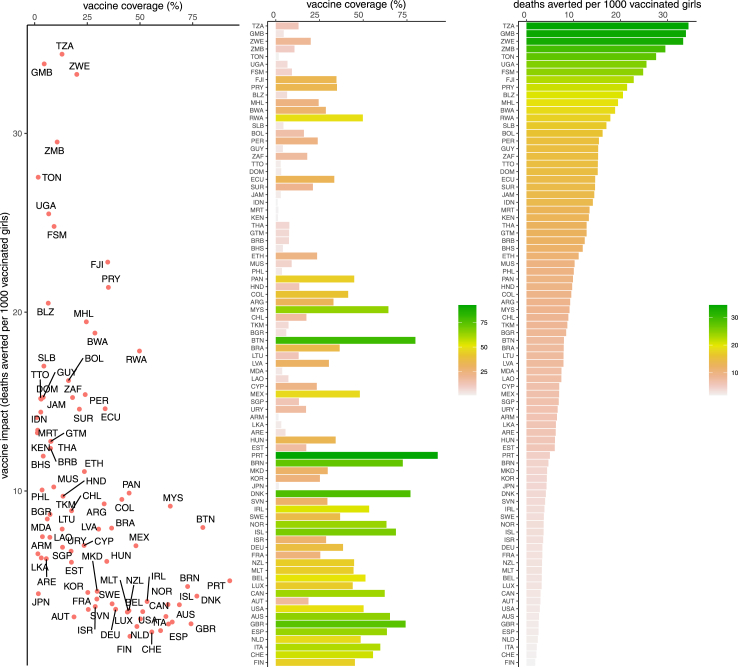
**HPV vaccination coverage and impact.** HPV vaccine coverage (average during 2010–22) and impact (deaths averted per 1000 vaccinated girls) among 84 countries. Left panel presents vaccine coverage versus impact, the middle panel presents vaccine coverage, and the right panel presents vaccine impact among the 84 countries (ranked from high to low vaccine impact). Countries are referred to by ISO-3 codes. ISO3 code, International Organization for Standardization three-letter country code; HPV, human papillomavirus.

In the WUENIC dataset, HPV vaccination coverage estimates were available for 8 countries in 2010 and for 90 countries in 2022. Coverage data were missing for some countries during 2010–22 where HPV vaccination was available. Fig. 1 (middle panel) shows the average coverage of HPV vaccination (last dose of HPV vaccination by age 15 years among females) among 84 countries during 2010–22. The average coverage during 2010–22 varied from 1% in Indonesia to 93% in Portugal, the former likely representing private sector purchases. HPV vaccine was recently introduced into the national programme in Indonesia in 2020 with a coverage of 3% which has since increased to 6% in 2022.

We estimated the lifetime health impact of HPV vaccination on cervical cancer burden in terms of deaths, cases, and DALYs averted per 1000 vaccinated girls among the vaccinated cohorts of 2010–22 in 84 countries (Fig. 1 and Table 1, plus Appendix Figures A1-2). The health impact of HPV vaccination varied across the 84 countries ranging from Switzerland to Tanzania at 2 to 34 deaths, 4 to 47 cases, and 40 to 735 DALYs averted per 1000 vaccinated adolescent girls over the lifetime of the vaccinated cohorts of 2010–22.

**Table 1:** HPV vaccination coverage and impact.

Country	ISO3 code	WHO region	Income level	HPV vaccine coverage (%)	Deaths averted per 1000 vaccinated girls	Cases averted per 1000 vaccinated girls	DALYs averted per 1000 vaccinated girls
Tanzania	TZA	Africa	Lower middle income	13	34	47	735
Gambia	GMB	Africa	Low income	5	34	38	466
Zimbabwe	ZWE	Africa	Lower middle income	20	33	44	588
Zambia	ZMB	Africa	Lower middle income	11	30	42	569
Tonga	TON	Western Pacific	Upper middle income	2	28	33	525
Uganda	UGA	Africa	Low income	7	25	32	515
Micronesia (Federated States of)	FSM	Western Pacific	Lower middle income	9	25	30	527
Fiji	FJI	Western Pacific	Upper middle income	35	23	28	420
Paraguay	PRY	Americas	Upper middle income	35	21	29	443
Belize	BLZ	Americas	Upper middle income	6	21	21	431
Marshall Islands	MHL	Western Pacific	Upper middle income	25	19	25	351
Botswana	BWA	Africa	Upper middle income	29	19	28	351
Rwanda	RWA	Africa	Low income	50	18	23	378
Solomon Islands	SLB	Western Pacific	Lower middle income	4	17	24	381
Bolivia	BOL	Americas	Lower middle income	16	16	24	360
Peru	PER	Americas	Upper middle income	24	15	22	299
Guyana	GUY	Americas	High income	4	15	24	304
South Africa	ZAF	Africa	Upper middle income	18	15	26	343
Trinidad & Tobago	TTO	Americas	High income	3	15	23	321
Dominican Republic	DOM	Americas	Upper middle income	3	15	18	275
Ecuador	ECU	Americas	Upper middle income	33	15	21	259
Suriname	SUR	Americas	Upper middle income	21	15	21	334
Jamaica	JAM	Americas	Upper middle income	3	14	20	309
Indonesia	IDN	South-East Asia	Upper middle income	1	14	20	293
Mauritania	MRT	Africa	Lower middle income	1	13	18	256
Kenya	KEN	Africa	Lower middle income	1	13	19	289
Thailand	THA	South-East Asia	Upper middle income	8	13	19	267
Guatemala	GTM	Americas	Upper middle income	8	13	18	251
Barbados	BRB	Americas	High income	8	12	18	250
Bahamas	BHS	Americas	High income	4	12	16	282
Ethiopia	ETH	Africa	Low income	24	11	14	240
Mauritius	MUS	Africa	Upper middle income	9	10	15	160
Philippines	PHL	Western Pacific	Lower middle income	4	10	14	183
Panama	PAN	Americas	High income	45	10	14	216
Honduras	HND	Americas	Lower middle income	13	10	14	238
Colombia	COL	Americas	Upper middle income	41	10	14	191
Argentina	ARG	Americas	Upper middle income	33	9	14	247
Malaysia	MYS	Western Pacific	Upper middle income	64	9	11	169
Chile	CHL	Americas	High income	18	9	13	181
Turkmenistan	TKM	Europe	Upper middle income	7	9	13	210
Bulgaria	BGR	Europe	Upper middle income	6	8	15	199
Bhutan	BTN	South-East Asia	Lower middle income	80	8	12	212
Brazil	BRA	Americas	Upper middle income	37	8	13	182
Lithuania	LTU	Europe	High income	13	8	16	199
Latvia	LVA	Europe	High income	30	8	15	192
Moldova	MDA	Europe	Upper middle income	4	7	12	182
Laos	LAO	Western Pacific	Lower middle income	7	7	11	149
Cyprus	CYP	Europe	High income	23	7	5	118
Mexico	MEX	Americas	Upper middle income	48	7	12	148
Singapore	SGP	Western Pacific	High income	13	7	9	132
Uruguay	URY	Americas	High income	17	7	10	165
Armenia	ARM	Europe	Upper middle income	2	6	9	144
Sri Lanka	LKA	South-East Asia	Lower middle income	3	6	10	145
United Arab Emirates	ARE	Eastern Mediterranean	High income	6	6	8	143
Hungary	HUN	Europe	High income	34	6	14	145
Estonia	EST	Europe	High income	17	6	17	143
Portugal	PRT	Europe	High income	93	5	10	114
Brunei	BRN	Western Pacific	High income	73	5	14	141
North Macedonia	MKD	Europe	Upper middle income	30	4	6	103
South Korea	KOR	Western Pacific	High income	25	4	8	78
Japan	JPN	Western Pacific	High income	2	4	12	108
Denmark	DNK	Europe	High income	77	4	9	79
Slovenia	SVN	Europe	High income	30	4	6	85
Ireland	IRL	Europe	High income	53	4	9	94
Sweden	SWE	Europe	High income	37	4	9	70
Norway	NOR	Europe	High income	63	4	9	68
Iceland	ISL	Europe	High income	69	4	6	116
Israel	ISR	Europe	High income	29	4	5	75
Germany	DEU	Europe	High income	38	3	7	76
France	FRA	Europe	High income	25	3	7	80
New Zealand	NZL	Western Pacific	High income	45	3	5	74
Malta	MLT	Europe	High income	44	3	4	64
Belgium	BEL	Europe	High income	51	3	7	71
Luxembourg	LUX	Europe	High income	44	3	6	69
Canada	CAN	Americas	High income	62	3	5	72
Austria	AUT	Europe	High income	19	3	6	66
United States	USA	Americas	High income	50	3	6	71
Australia	AUS	Western Pacific	High income	65	3	5	59
United Kingdom	GBR	Europe	High income	74	3	7	65
Spain	ESP	Europe	High income	64	3	5	62
Netherlands	NLD	Europe	High income	49	2	6	50
Italy	ITA	Europe	High income	60	2	6	56
Switzerland	CHE	Europe	High income	56	2	4	40
Finland	FIN	Europe	High income	45	2	4	41

The average coverage of HPV vaccination among 84 countries^a^ during 2010–22 and lifetime health impact measured by deaths, cases, and DALYs averted per 1000 vaccinated girls. Countries are ordered from high to low vaccine impact in terms of deaths averted per 1000 vaccinated girls. Regions are based on grouping of WHO member states into six regions—Africa, Americas, South-East Asia, Europe, Western Pacific, and Eastern Mediterranean. Income levels are based on the World Bank Group assignment of the world's economies to four income groups—high, upper-middle, lower-middle, and low. ISO3 code, International Organization for Standardization three-letter country code; WHO, World Health Organization; HPV, human papillomavirus; DALYs, disability-adjusted life years.

a HPV vaccination coverage for Andorra, Cook Islands, Palau, San Marino, Seychelles, and Saint Vincent and the Grenadines are available from the UNICEF immunisation data portal. However, these 6 countries were excluded in this study due to lack of data on other key inputs (such as demography) to the HPV vaccine impact model (PRIME).

### Inequities in HPV vaccination coverage and impact

**Fig. 2:**
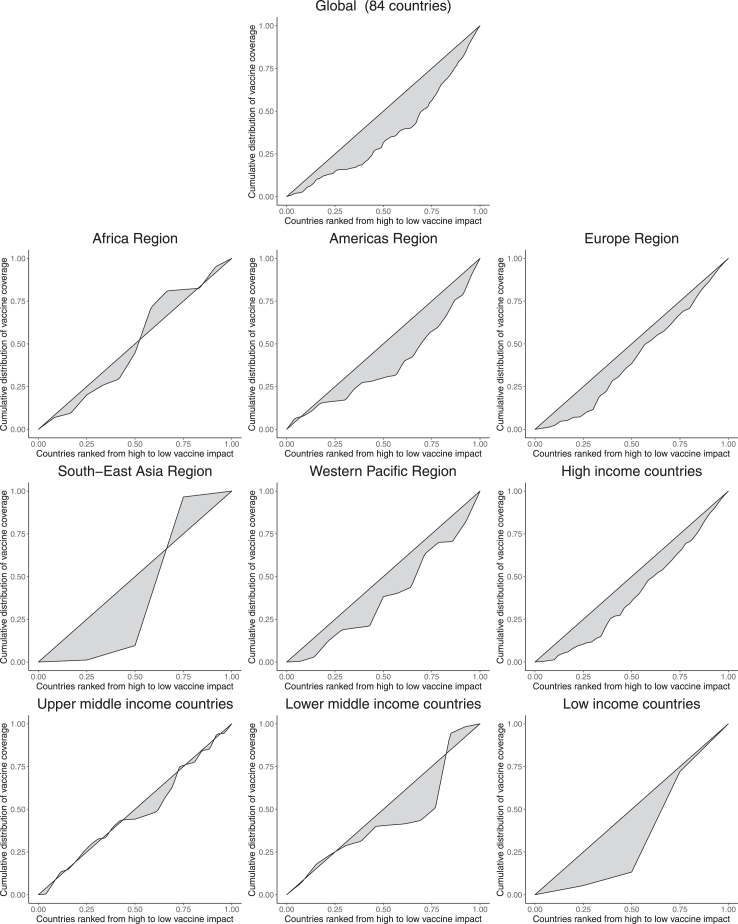
**Inequities in HPV vaccination coverage and impact (deaths averted per 1000 vaccinated girls).** The concentration curves illustrate the inequities in HPV vaccine coverage and impact among the countries at the global, regional, and income levels. Countries are ranked from high to low vaccine impact (same as countries ranked by high to low burden of cervical cancer), and vaccine impact is measured by deaths averted per 1000 vaccinated girls. Since the United Arab Emirates was the only country from the Eastern Mediterranean region in our study, the concentration curve for this region is not applicable. HPV, human papillomavirus.

Fig. 2 and Table 2 (plus Appendix Figure A3) show the concentration curves and indices to estimate the inequities in the average coverage of HPV vaccination among the 84 countries during 2010–22 at the global, regional, and income levels.

**Table 2:** Concentration indices.

Countries (Global, WHO regions, and World Bank income levels)	Number of countries	Concentration indices (Concentration index and 95% confidence interval)		
		Impact by deaths averted per 1000 vaccinated girls	Impact by cases averted per 1000 vaccinated girls	Impact by DALYs averted per 1000 vaccinated girls
Global	84	0.33 (0.27, 0.40)	0.31 (0.24, 0.38)	0.33 (0.26, 0.40)
Africa Region	12	0 (−0.21, 0.21)	0.03 (−0.17, 0.23)	−0.05 (−0.24, 0.15)
Americas Region	23	0.38 (0.22, 0.53)	0.39 (0.23, 0.55)	0.41 (0.26, 0.56)
Europe Region	30	0.31 (0.19, 0.43)	0.16 (0.04, 0.29)	0.32 (0.20, 0.44)
South-East Asia Region	4	0.25 (−0.45, 0.95)	0.25 (−0.45, 0.95)	0.25 (−0.45, 0.95)
Western Pacific Region	14	0.36 (0.14, 0.57)	0.34 (0.12, 0.55)	0.34 (0.13, 0.56)
Eastern Mediterranean Region	1	–	–	–
High income countries	41	0.35 (0.25, 0.45)	0.26 (0.15, 0.37)	0.34 (0.24, 0.44)
Upper middle income countries	26	0.06 (−0.13, 0.24)	0.09 (−0.11, 0.29)	0.12 (−0.07, 0.30)
Lower middle income countries	13	0.09 (−0.30, 0.48)	0.09 (−0.30, 0.48)	0.04 (−0.31, 0.39)
Low income countries	4	0.55 (0.17, 0.94)	0.55 (0.17, 0.94)	0.53 (0.17, 0.89)

The concentration indices measure the inequities in HPV vaccination coverage and impact among the countries at the global, regional, and income levels. Vaccine impact is estimated by deaths, cases, and DALYs averted per 1000 vaccinated girls. Since the United Arab Emirates was the only country from the Eastern Mediterranean region in our study, the concentration index for this region is not applicable. HPV, human papillomavirus; DALYs, disability-adjusted life years; WHO, World Health Organization.

At the global level among the 84 countries, when vaccine impact was measured by deaths averted per 1000 vaccinated girls, the concentration index for the distribution of average coverage during 2010–22 was 0.33 (95% CI: 0.27, 0.40). This indicates a pro-advantaged distribution (regressive, i.e. favouring low impact countries). The concentration indices for the distribution of vaccine coverage among the 84 countries were 0.31 (0.24, 0.38) and 0.33 (0.26, 0.40) when vaccine impact is measured in terms of cases and DALYs averted per 1000 vaccinated girls respectively. This further indicates a pro-advantaged regressive distribution favouring low impact countries.

By WHO regions, HPV vaccination coverage varied significantly across the 84 countries. In the Americas, Europe, and Western Pacific regions, encompassing 23, 30, and 14 countries respectively, the concentration indices were 0.38 (0.22, 0.53), 0.31 (0.19, 0.43), and 0.36 (0.14, 0.57) respectively. This highlights significant inequities in HPV vaccination coverage within each of these three regions. The coverage distribution was pro-advantaged and regressive, favouring countries with relatively lower impact of vaccination. The concentration indices were 0 (−0.21, 0.21) and 0.25 (−0.45, 0.95) among the 12 and 4 countries in the Africa and South-East Asia regions respectively. The concentration curves present mixed patterns in these two regions with inequitable coverage at higher levels of vaccine impact and equitable coverage at lower levels of vaccine impact. Since the United Arab Emirates was the only country from the Eastern Mediterranean region in our study, we precluded inferences for this region.

By World Bank income levels, HPV vaccination coverage also varied significantly across the 84 countries. In the 41 high-income countries, the concentration index was 0.35 (0.25, 0.45), highlighting significant inequities in HPV vaccination coverage. The coverage distribution is pro-advantaged and regressive, favouring countries with lower vaccine impact. In comparison, the 26 upper middle income countries present a relatively equitable landscape with a concentration index of 0.06 (−0.13, 0.24). In the 13 lower middle income countries, equitable coverage is observed at the highest and lowest levels of vaccine impact, but inequity persists at mid-levels of impact with a concentration index of 0.09 (−0.30, 0.48). The four low income countries had relatively high levels of inequity in coverage with a concentration index of 0.55 (0.17, 0.94). Among these four low income countries, HPV vaccine coverage is relatively lower in Gambia, Uganda, and Ethiopia in comparison to Rwanda while vaccine impact is relatively lower in Ethiopia.

## Discussion

Our findings suggested that countries with a relatively higher cervical cancer burden and thereby a relatively higher need for HPV vaccination had relatively lower coverage during 2010–22. Further, many countries with higher cervical cancer burden are yet to introduce HPV vaccination in their national immunisation programmes. The current state of HPV vaccine introduction, scale-up, and coverage after more than 17 years since the first HPV vaccines being available since 2006 highlights the persistent challenges to equitable access to HPV vaccines and more broadly on fair access to vaccines in general, especially in low- and middle-income countries.^26^ As of July 2023 among the 194 WHO member countries, 131 countries have introduced HPV vaccination in their national immunisation programmes while three countries have partially included and 60 countries have not included yet.^27^

There were significant inequities in HPV vaccination coverage within the Americas, Europe, and Western Pacific regions, and in high- and low-income countries with a pro-advantaged and regressive distribution favouring countries with lower vaccine impact. Japan was amongst the countries with low coverage (less than 2% on average during 2010–22)—lowest among high-income countries, second lowest in the Western Pacific region, and sixth lowest among the 84 countries. Thereby, factors beyond economic resources such as vaccine confidence and acceptance among adolescent girls and parents/caregivers are important to be addressed by evidence-based communication and social mobilisation for effective implementation of HPV vaccination programmes.^28^^,^^29^

Since the WHO SAGE favourable recommendation in 2022 on the one-dose regimen and inference that the one-dose regimen likely provides comparable protection to that of a two-dose regimen among adolescent girls, some countries including Australia and UK have shifted to the one-dose schedule for adolescent girls from 2023.^30^^,^^31^ This new recommendation provides a highly favourable pathway to lower the programmatic barriers in HPV vaccine delivery as well as inequities in HPV vaccination coverage within and between countries, thereby potentially accelerating progress towards cervical cancer elimination as a public health problem. This change in policy is also timely with projected increases in HPV vaccine supply and thereby provides a good opportunity to scale up coverage in countries with current HPV vaccination programmes and extend access to countries with partial inclusion or no introduction yet.^32^

Our study has limitations. Since coverage refers to the last dose of HPV vaccination by age 15 years among females, vaccination could have occurred at 14-years of age or earlier. However, we used the conservative assumption of vaccination at 14-years of age among adolescent girls. Coverage data are missing for some countries during 2010–22 where HPV vaccination was available. HPV vaccination impact would be relatively higher than estimated by PRIME due to the conservative assumptions of the analysis—indirect (herd) effects are excluded, vaccine has no protective effect when administered post-sexual debut, cervical cancer incidence, prevalence, and mortality estimates remain constant, and cross-protection (or direct protection of the nonavalent vaccine against HPV types 31/33/45/52/58) against high-risk HPV types apart from HPV types 16/18 are not considered. These assumptions are likely to underestimate the inequity between countries, since relaxing them would further increase vaccine impact in high coverage countries, especially those using the nonavalent vaccine and/or gender-neutral vaccination. Since we have used HPV vaccination coverage for only the adolescent girls and not boys in our modelling study, gender-neutral vaccination which is mostly provided by high-income and relatively low vaccine impact countries would further widen the inequities in HPV vaccination coverage and impact among the countries. We have not included HPV vaccination cost nor differential pricing mechanisms and financing by country-income levels which affect access and have been exacerbated by the global shortage of HPV vaccines. Further, we have not considered the unique challenges faced by middle-income countries that are caught in the middle in regard to financing, procurement and production, registration and marketing authorisation, and distribution and uptake of HPV vaccines.

In conclusion, there are persistent inequities in HPV vaccination coverage across countries. Countries with a relatively higher cervical cancer burden and thereby with a higher need for HPV vaccination have relatively lower coverage of HPV vaccination. The recent WHO SAGE recommendation for a one-dose regimen provides an ideal moment to address these inequities by lowering the barriers on the programmatic, logistic and financial fronts in comparison to a two-dose or three-dose regimen. We recommend a globally coordinated initiative to address these inequities in HPV vaccination between and within countries, thereby delivering on a fair and equitable access to HPV vaccination globally. This imperative on HPV vaccine equity will have to be complemented with evidence on cervical cancer burden, health benefits of HPV vaccination, values and preferences of target population, acceptability to stakeholders, resources use, and feasibility to facilitate introduction and scale-up of HPV vaccination in all countries.^28^

## Contributors

KA and MJ conceptualised the study. KA conducted the equity impact analysis and wrote the original draft. KA and KJY verified the underlaying data. All authors contributed to interpretation of results, critical review and editing of the manuscript, and have approved the final version. The authors alone are responsible for the views expressed in this article and they do not necessarily represent the decisions, policy or views of their affiliated organisations. All authors had full access to data in the study, and final responsibility for the decision to submit for publication.

## Data sharing statement

The analysis software is publicly accessible and all data were from secondary sources in the public domain. The program code and data for the equity impact analysis in this study are publicly accessible online at https://github.com/vaccine-impact/hpv_vaccine_equity. All analyses were done using the R statistical software (version 4.1.2). The R package of PRIME is accessible at https://github.com/lshtm-vimc/prime. The HPV vaccination coverage data are publicly available from the UNICEF immunisation data portal at https://data.unicef.org/resources/dataset/immunization.

## Editor note

The Lancet Group takes a neutral position with respect to territorial claims in published maps and institutional affiliations.

## Declaration of interests

We declare no competing interests.

## Appendix A Supplementary data

Supplementary Information

Supplementary_data_burden_impact
