# Supplementary material for: Equity impact of HPV vaccination on lifetime projections of cervical cancer burden among cohorts in 84 countries by global, regional, and income levels, 2010–22: a modelling study
Source: eClinicalMedicine. 2024 Mar 11;70:102524. doi: 10.1016/j.eclinm.2024.102524 (PMC11056390; doi:10.1016/j.eclinm.2024.102524)

Supplementary information

A1. Model summary

The Papillomavirus Rapid Interface for Modelling and Economics (PRIME) is a static, proportional impact model that can estimate the impact of HPV vaccination on cervical cancer deaths, cases, and disability-adjusted life years as well as the cost-effectiveness of vaccination programmes at the global, regional, and national levels [1, 2]. It has been used to support vaccine recommendations by WHO, as well as individual countries. It has been validated against published studies using HPV vaccine economic models set in LMICs [1]. It was also endorsed by the WHO's expert advisory committee, the Immunization and Vaccines Implementation Research Advisory Committee (IVIR-AC) to provide a conservative estimate of the cost effectiveness of vaccinating girls prior to sexual debut.

Data inputs include country and age-specific cervical cancer incidence, prevalence, and mortality among females. The model estimates vaccination impact in terms of reduction in age-dependent incidence of cervical cancer and mortality in direct proportion to vaccine efficacy against HPV 16/18, vaccine coverage, and HPV type distribution. It assumes that vaccinating girls prior to infection with HPV types 16 and 18 fully protects them from developing cervical cancer caused by HPV 16 and 18, in accordance with vaccine trials.

HPV vaccine impact estimation

HPV vaccine impact on cervical cancer burden (incidence, prevalence and mortality) averted is calculated as the proportional reduction in age-specific incidence, prevalence and mortality respectively in each country at single-year age intervals from 0-100 years.

cervical cancer *burden* averted at age *i* =

cervical cancer *burden* caused by all HPV genotypes at age *i* pre-vaccination *

country-specific proportion of cervical cancer caused by HPV 16/18 *

vaccine coverage at prior age of vaccination *v* ($v \leq i$) *

vaccine efficacy against HPV 16/18 *

proportion of female population that has not experienced sexual debut by age of vaccination *v*

Cervical cancer burden calculation

Incidence, prevalence and mortality for a given age and specific country are defined as follows:

- Incidence at age *a* in country *c* is the proportion in the specified population who were diagnosed with cervical cancer (at age *a*).

- Prevalence at age *a* in country *c* is the proportion in the specified population who had been diagnosed with cervical cancer within the last 5 years and are still alive.
- Mortality at age *a* in country *c* is the proportion in the specified population who died due to cervical cancer.

$$\text{DALY (disability-adjusted life year)} = \text{YLD (years lived with disability)} + \text{YLL (years of life lost to due to premature mortality)}$$

Morbidity was attributed to the age of prevalence using disability weights and durations for different phases of cervical cancer.

$$\begin{aligned}\text{YLD}_{\text{a, c}} = \ & \text{incidence}_{a,c} * (\text{duration}_{\textit{diagnosis phase}} * \text{disability weight}_{\textit{diagnosis phase}}) + \\ & \text{prevalence}_{a,c} * (\text{disability weight}_{\textit{control phase}}) + \\ & \text{mortality}_{a,c} * (\text{duration}_{\textit{metastatic phase}} * \text{disability weight}_{\textit{metastatic phase}} + \\ & \quad \text{duration}_{\textit{terminal phase}} * \text{disability weight}_{\textit{terminal phase}})\end{aligned}$$

Mortality impact was based on the remaining life expectancy at the age of death (*a*) for a given calendar year (*y*) and country (*c*).

$$\text{YLL}_{\text{a, c, y}} = \text{mortality}_{a,c,y} * \text{remaining life expectancy}_{a,c,y}$$

References (model summary)

1. Jit M, Brisson M, Portnoy A, Hutubessy R. Cost-effectiveness of female human papillomavirus vaccination in 179 countries: a PRIME modelling study. Lancet Global Health 2014; 2: e406-14.
2. Abbas KM, van Zandvoort K, Brisson M, Jit M. Effects of updated demography, disability weights, and cervical cancer burden on estimates of human papillomavirus vaccination impact at the global, regional, and national levels: a PRIME modelling study. Lancet Global Health 2020; 8: e536–44.

Figures (appendix)

Figure A1. Health impact of HPV vaccination. Lifetime health impact of HPV vaccination on the cervical cancer burden (caused by high-risk HPV 16/18 genotypes) in terms of deaths, cases, and DALYs averted per 1000 vaccinated girls (last dose of HPV vaccination by age 15 years among females) among the vaccinated cohorts of 2010–22 in 84 countries.

(a) Health impact of HPV vaccination (deaths averted per 1000 vaccinated girls)

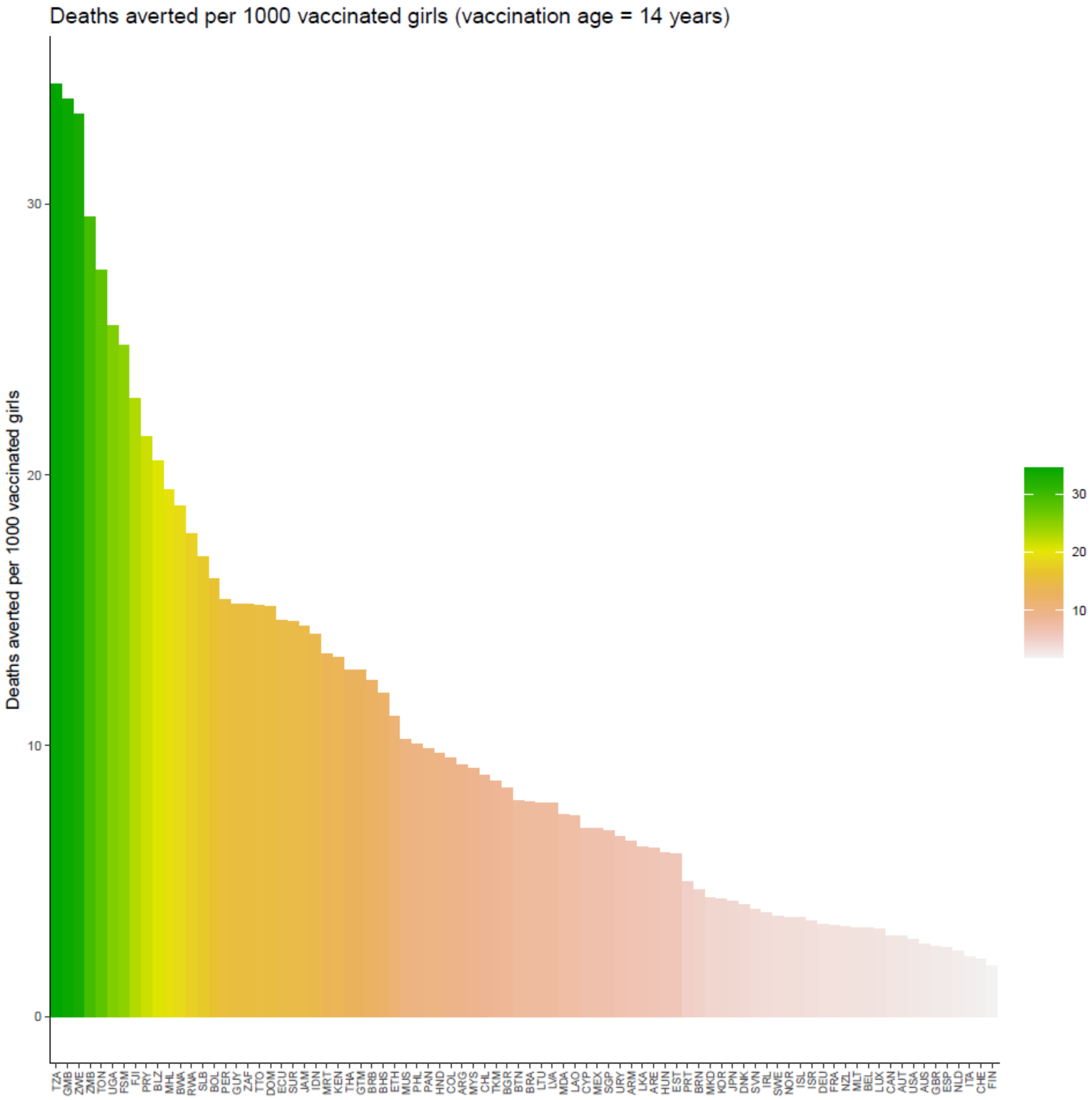

(b) Health impact of HPV vaccination (cases averted per 1000 vaccinated girls)

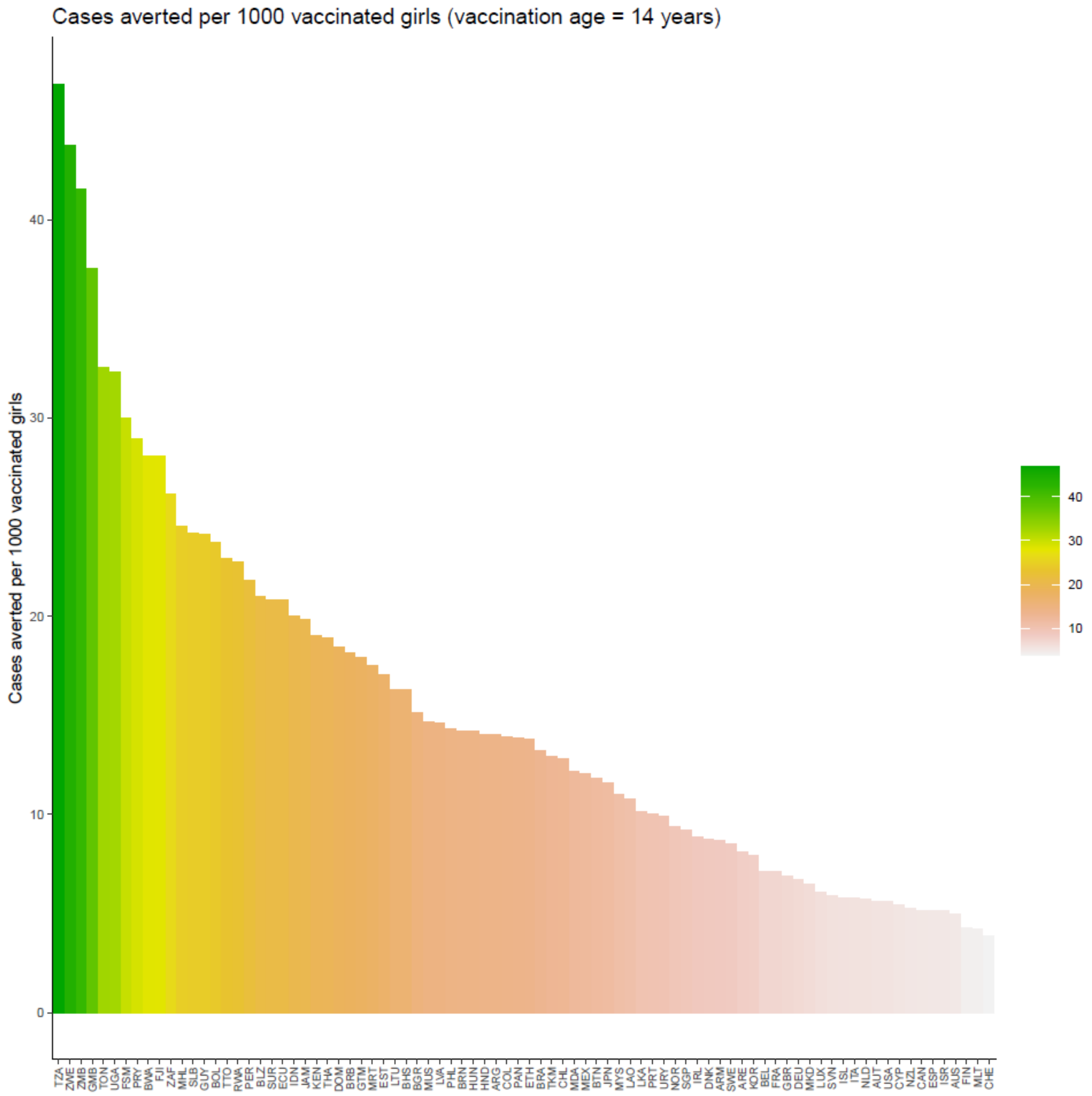

(c) Health impact of HPV vaccination (DALYs averted per 1000 vaccinated girls)

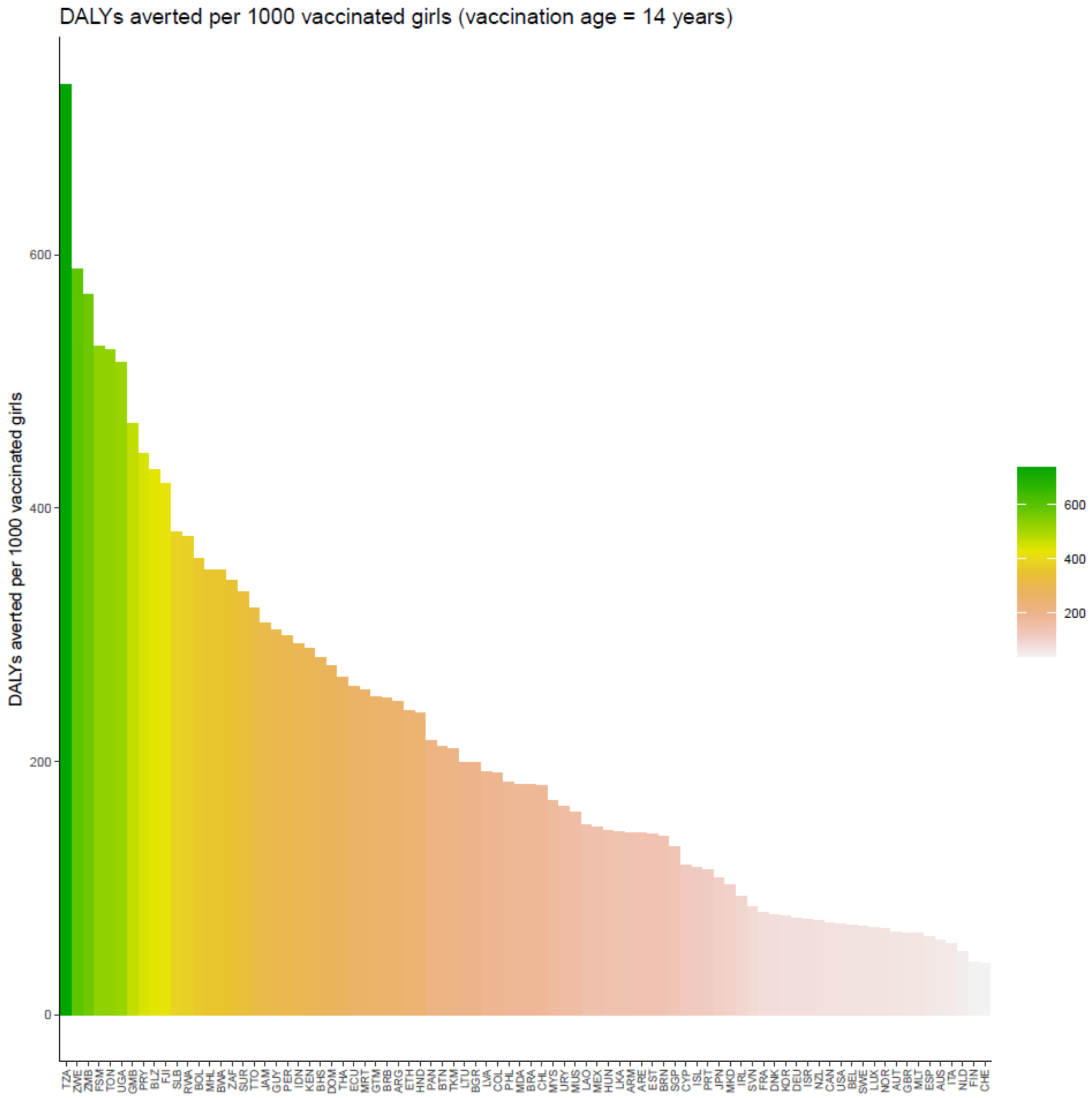

Figure A2. HPV vaccination coverage and impact. The scatter plot shows HPV vaccine coverage (average percentage during 2010–22) and impact (deaths, cases, and DALYs averted per 1000 vaccinated girls) among the 84 countries.

(a) HPV vaccination coverage and impact (deaths averted per 1000 vaccinated girls)

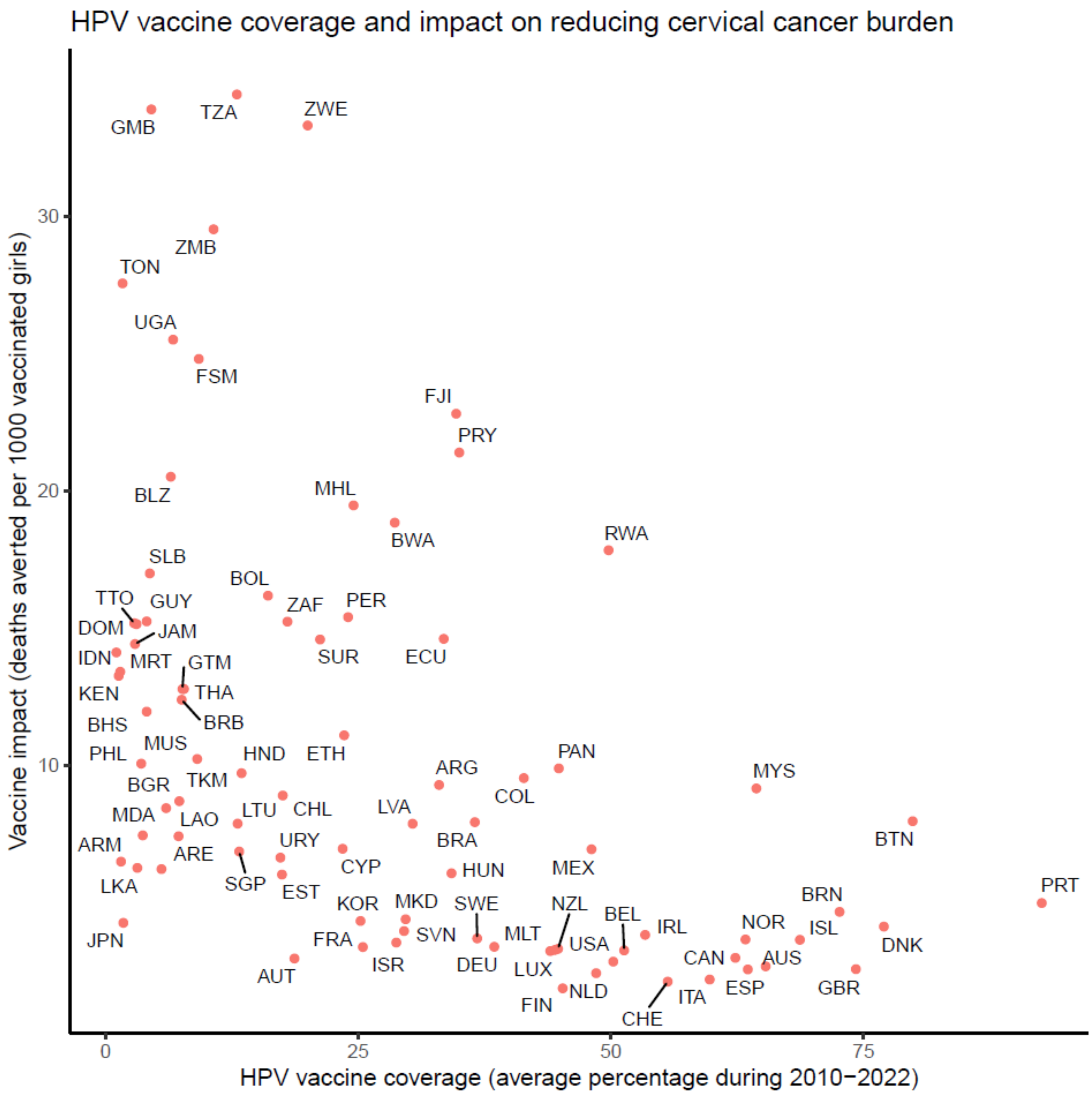

(b) HPV vaccination coverage and impact (cases averted per 1000 vaccinated girls)

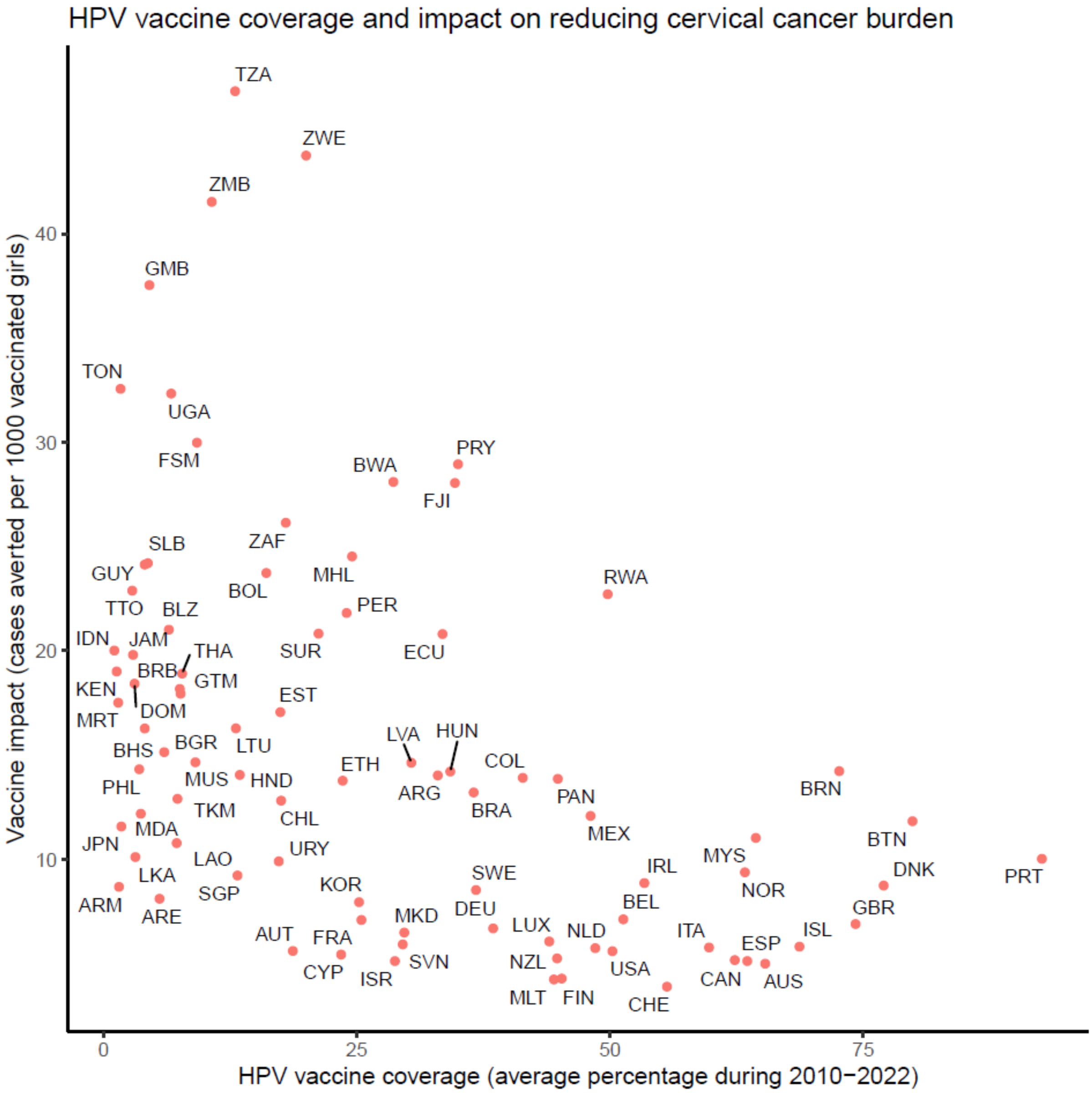

(c) HPV vaccination coverage and impact (DALYs averted per 1000 vaccinated girls)

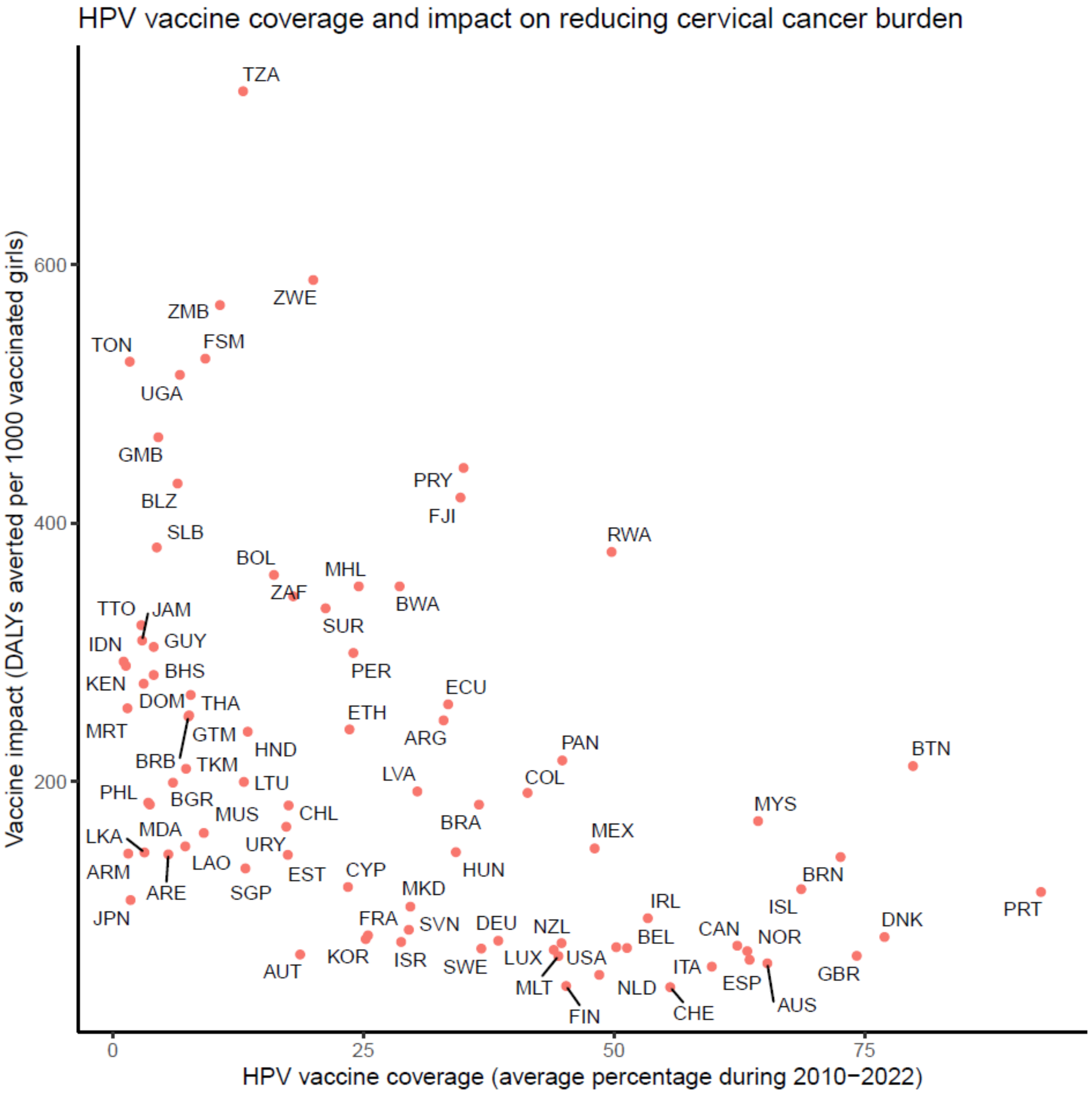

Figure A3. Inequities in HPV vaccination coverage and impact. The concentration curves illustrate the inequities in HPV vaccine coverage and impact among the countries at the global, regional levels, and income levels. Countries are ranked from high to low vaccine impact (same as countries ranked by high to low burden of cervical cancer), and vaccine impact is measured by cases and DALYs averted per 1000 vaccinated girls. Since the United Arab Emirates was the only country from the Eastern Mediterranean region in our study, the concentration curve for this region is not applicable.

(a) Inequities in HPV vaccination coverage and impact (cases averted per 1000 vaccinated girls)

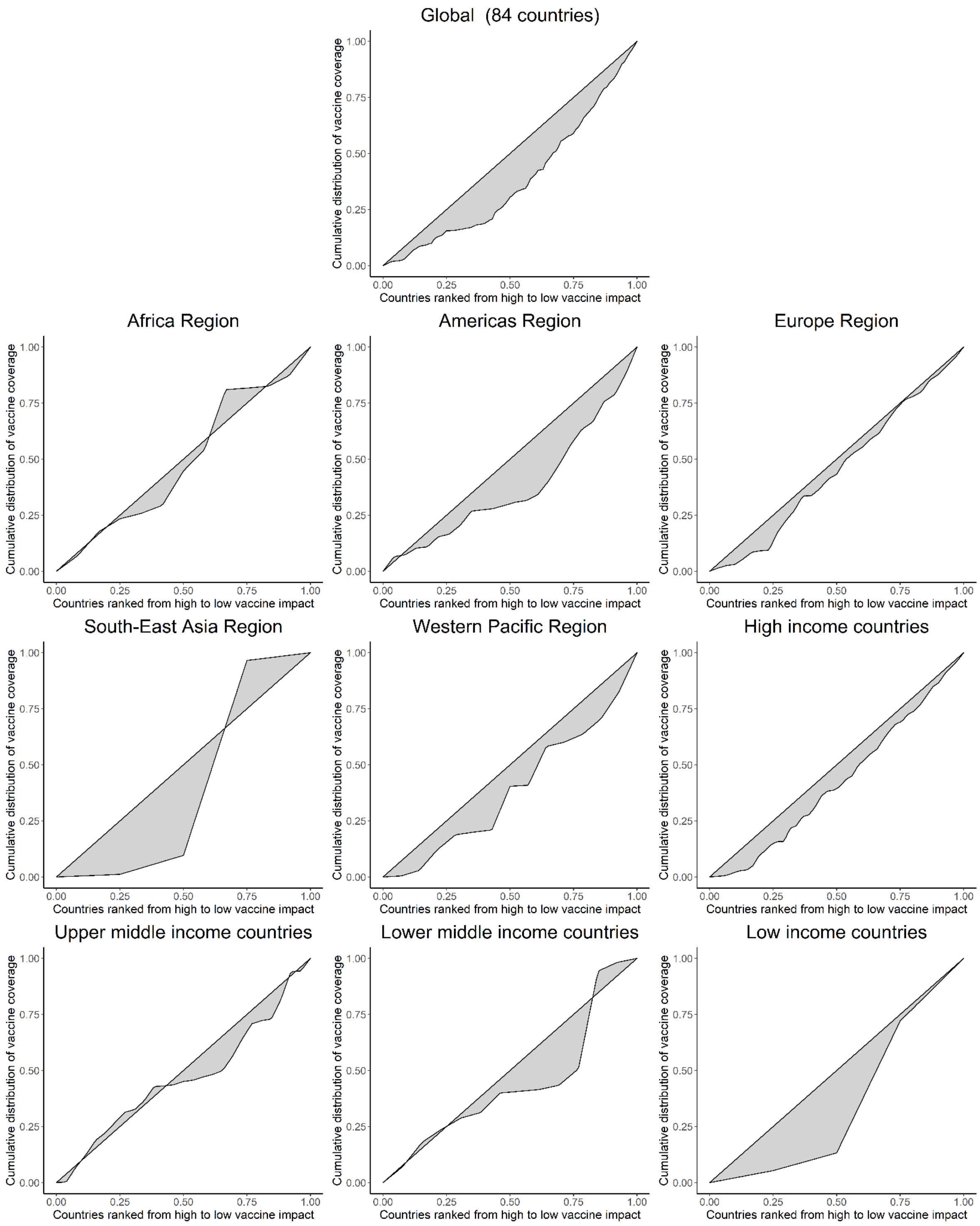

(b) Inequities in HPV vaccination coverage and impact (DALYs averted per 1000 vaccinated girls)

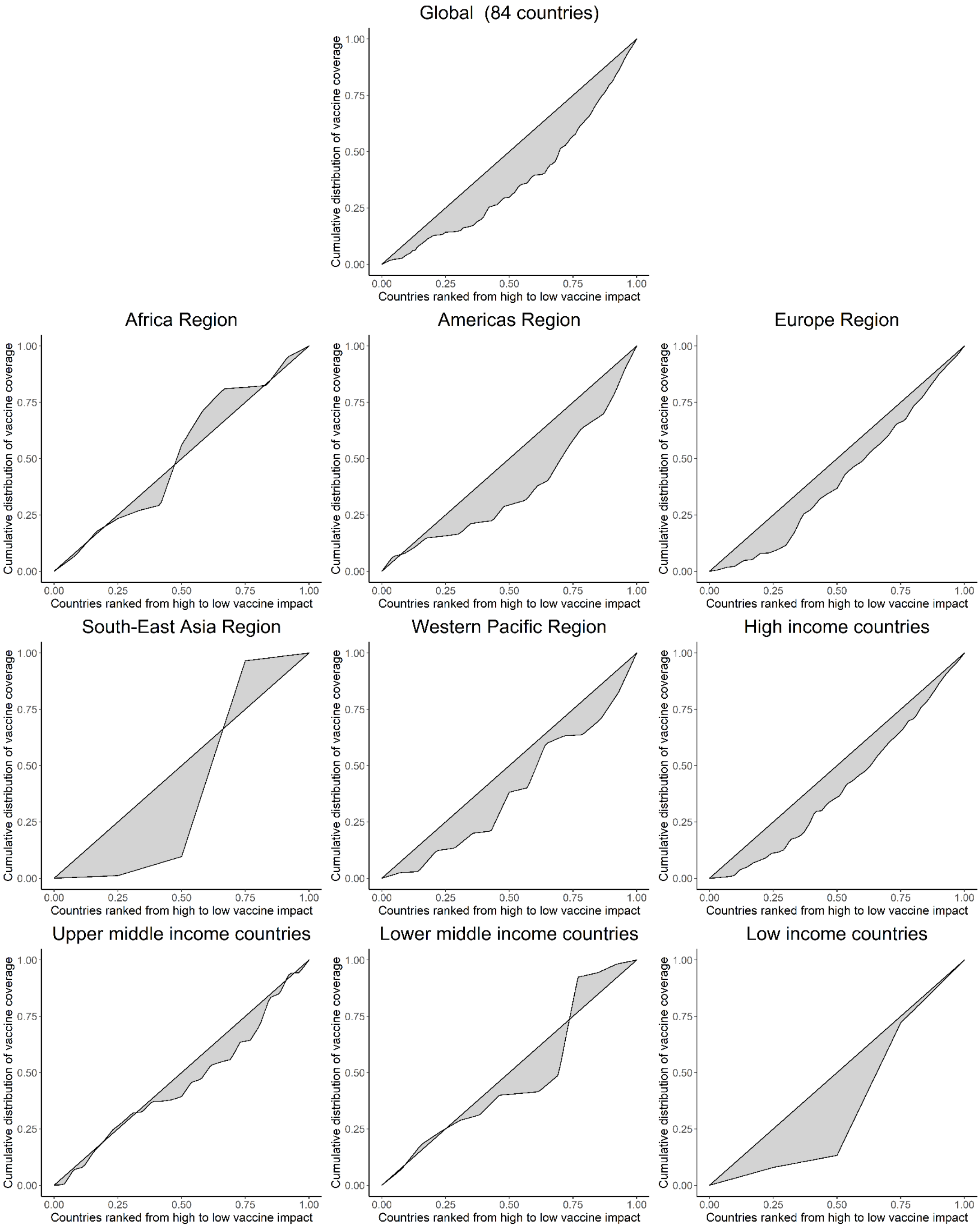

Supplement: Supplementary Information [file mmc1.pdf]
